# Premenstrual Disorders: Prevalence and Associated Factors in a Sample of Iranian Adolescents

**DOI:** 10.5812/ircmj.2084

**Published:** 2013-08-05

**Authors:** Mahin Delara, Hamed Borzuei, Ali Montazeri

**Affiliations:** 1 Social Determinants of Health Research Center, Faculty of Health, Sabzevar University of Medical Sciences, Sabzevar, IR Iran; 2 Research Development Center, Crez Co., Tehran, IR Iran; 3 Mental Health Research Group, Health Metrics Research Centre, Iranian Institute for Health Sciences Research, ACECR, Tehran, IR Iran

**Keywords:** Premenstrual Syndrome, Depressive Disorder, Prevalence, Dysmenorrhea

## Abstract

**Background:**

Premenstrual disorders usually refer to Premenstrual Syndrome (PMS) and Premenstrual Dysphoric Disorder (PMDD). This study was designed to find out the frequency of premenstrual disorders and evaluate the associated factors in a sample of Iranian adolescents.

**Objectives:**

This study was conducted to investigate the frequency of premenstrual disorders (PMS and PMDD) based on Premenstrual Assessment Scale (PAS) and also to determine the association of some demographic and menstrual characteristics with these disorders in adolescent girls.

**Patients and Methods:**

This was a cross sectional study. A sample of adolescent school girls aged between 14 and 19 years were included in the study. Diagnostic assessments were based on Premenstrual Assessment Scale (PAS). The data were analyzed in a descriptive fashion and were compared among subgroups of the study sample. In addition, demographic and menstrual factors associations with premenstrual disorders were assessed.

**Results:**

In all 1379 female students were included in the study. About 99.5 % of the students reported at least one premenstrual symptom. Of these, 66.3% was mild, 31.4% moderate and 2.3% severe. A total of 814 girls (59%) met the diagnostic criteria for premenstrual dysphoric disorder (PMDD). Most frequently reported symptoms were back pain, lethargy, fatigue and anxiety. Early menarche, lower education was associated with higher scores on PAS.

**Conclusion:**

Premenstrual disorders are common in adolescent girls. Preventive and treatment strategies are highly recommended.

## 1. Background

Premenstrual disorders usually refer to Premenstrual Syndrome (PMS) and Premenstrual Dysphoric Disorder (PMDD) are a set of physical, cognitive, affective, and behavioral symptoms that occur periodically during the luteal phase of the menstrual cycle and resolve at or within a few days of the onset of menstruation (1, 2). The typical symptoms, include irritability, anger, mood swings, depression, tension, anxiety, abdominal bloating, breast pain and fatigue.(3) Women with severe symptoms usually report impairment in their interpersonal or workplace functioning (3, 4). Although the true prevalence of premenstrual disorders is unknown, based on the various diagnostic criteria, the reports of the incidence of PMS vary from 0% to 60%. Premenstrual disorders start to be problematic in the adolescent years and decline in the climacteric phase of a woman’s life (3). During the teen years, premenstrual symptoms can complicate the process of puberty, interpersonal relationships, social and educational performance. They can also result in poor self-esteem, a sense of dissatisfaction, inadequacy and unhealthy life style (5). Previous studies have also shown that women with premenstrual disorders have a poor health-related quality of life (6-11).

Numerous studies have been conducted to elucidate underlying factors contributing to this disorder including various socioeconomic, biological, life style, behavioral and social variables(12-15). Finally, the relevance of factors such as age, temperament, anthropometric characteristics, reproductive and menstrual history, has been assessed (16, 17). Many studies had reported premenstrual disorders prevalence in Iranian population based on different diagnostic criteria but no agreed or validated generic diagnostic instrument compatible to Iranian culture was used in these studies (18, 19).

## 2. Objectives

This study was conducted to investigate the frequency of premenstrual disorders (PMS and PMDD) based on Premenstrual Assessment Scale (PAS) and also to determine the association of some demographic and menstrual characteristics with these disorders in adolescent girls.

## 3. Patients and Methods

### 3.1. The Study Sample and Design

A cross sectional study was conducted in Sabzevar high schools. Sabzevar is one of the Khorasan province cities located in the east part of Iran. All of the students in these high schools were invited to participate in the research. Students with a) amenorrhea, b) irregular menstrual cycles, c) menstrual cycles shorter than 21 days or longer than 35 days d) major medical and psychological problems and those e) receiving hormonal therapy or f) experiencing a catastrophe shortly before or during the study, were excluded from the research. In addition, students were excluded if they reported symptoms of psychological problems. This was indicated by the Symptom Checklist 90- Revised, SCL-90R. 20Scores greater than 63 were considered as the cut-off point for psychological disorder as recommended (20).

### 3.2. Premenstrual Symptoms Measure

Premenstrual symptoms were measured by using the Premenstrual Assessment Scale (PAS). PAS is a 32 item designed questionnaire with a scaling from zero (absence of symptoms) to 3 indicating the severity of the symptoms (0- no symptom, 1-mild, 2-moderate, 3-sever). This scale was developed and validated through an Iranian research. Content and construct validity assessed for this retrospective self-report scale indicated two underlying dimension, i.e., physical and psychological symptoms. The resultant questionnaire also demonstrated a coefficient alpha value of 0.924 and Pearson correlation coefficient of 0.84 corresponding to a very high level of internal consistency and test -re- test reliability (21).

PAS can also be used for the diagnosis of PMDD which is characterized by the presence of at least five symptoms, one of which must be depressed mood, hopelessness, humiliation, anxiety, tension, affect liability, eluding from people, marked anger, increased conflicts, irritability, exciting and restless which all are included in the PAS. The symptoms must occur in the late luteal phase and should not be a luteal exacerbation of an existing psychiatric condition that interferes with social activities or interpersonal relationships. Other symptoms are confusion, decreased interest in usual activities, concentration difficulties, lethargy, fatigability, impatience, no control on behavior, lack of energy, appetite change, overeating, food cravings, hypersomnia, insomnia, breast tenderness, breast swellings, flatulence, swelling of extremities, weight gain, headache, back or lower abdominal pain, joint or muscle pain (21). The students were asked to indicate which of the above symptoms had occurred one week before and after the menstruation during the previous year.

A questionnaire for collecting data on demographic and menstrual characteristics of the study sample was also used. This questionnaire consisted of 3 parts. Part 1 included questions about socio-demographic information such as age, marital status, parents’ employment, and economic status. Part 2 included questions about menstrual characteristics such as the severity of menstrual bleeding, the length of menstrual bleeding, menstrual cycle duration, the presence of dysmenorrhea and menarche age. Part 3 included questions about educational performance and frequency of absenteeism from school. The participants were asked to voluntarily answer the questions in their classrooms break time.

### 3.3. Statistical Analysis

The data were analyzed in a descriptive fashion. Mann-Whitney U test was used for comparison. Moreover a Spearman correlation analysis was used to assess any relation between the demographic and menstrual characteristics information provided by the students and the PAS scores. Finally, a multivariate regression analysis was performed to estimate the linear relation between dependent variables (PAS scores) and various independent variables which their statistically significant correlations were previously assessed. Chi square analysis was also used to determine the correlation between some qualitative variables and the presence of PMS/PMDD. Finally, a logistic regression analysis was performed to determine variables that contribute to PMS/PMDD in students.

## 4. Results

In all, 1722 female students were approached from 6 random selected high schools. Of these 1710 students (99.3%) met the inclusion criteria. Among these, 1379 (81%) returned the self-report questionnaire completed in all aspects. The mean age of participants was 15.76 ± 1.1 years (ranging from 14 to 19 years). The majority were single (91.3%) and 81.9 % were accommodated in school dormitories. The mean duration of menstrual bleeding was 6.4 ± 2.2 days and the mean age of menarche was 13.1 ± 1.1 years. Moderate menstrual bleeding was reported in 70.4 % of the respondents. The characteristics of the participants are shown in Table 1.

**Table 1.** The Characteristics of the Study Sample (n = 1379 adolescents)

	No (%)
**Age(year)**	
Mean (SD)	15.76 (1.02)
Range	14-19
**Marital status**	
Single	1260 (91.3)
Married	119 (8.7)
**Menarche age(year)**	
Mean (SD)	15 (1.1)
Range	9-19
**Amount of menstrual bleeding**	
Low	308 (22.3)
Moderate	971 (70.4)
Severe	100 (7.3)
**BMI, kg/m** ^**2**^	
< 19.8	663 (48.1)
19.8-26	604 (43.8)
26-29	51 (3.7)
29 <	61 (4.4)

From the total 1379 individual, 7 students were symptom free so the prevalence of PMS was calculated to be 99.5 % according to PAS. Among those 1372, 66.3 % of the respondents had mild PMS, 31.4 % experienced moderate PMS symptoms and 2.3 % suffered from severe PMS whereas 814 (59%) girls were diagnosed as having PMDD. The minimum score for total PAS items, was 1 and the maximum was 79 with a mean score 26.42 ± 16.6. Physical symptoms mean score was 10.2 ± 6.6 and the mean score for psychological symptoms was 16.33 ± 11.31. As demonstrated in Tables 2 and 3 the most reported physical symptoms were lower abdominal and back pain (89.7%) and lethargy was the major psychological complaint of respondents (80.4%). The lowest frequent symptom was swelling of extremities (11.3%). In 39.2 % of the students, the symptoms interfered with family relationships, 42.6% with educational performance and 40.6 % with social interactions.

**Table 2.** Percentage of the Sample Reporting Various Premenstrual Psychological Symptoms from the Premenstrual Assessment Scale (n = 1379)

Item	No symptom, No (%)	Mild, No (%)	Moderate, No (%)	Severe, No (%)
**Depressive Mood**	695 (50.4)	356 (25.8)	283 (20.5)	45 (3.3)
**Hopelessness**	972 (70.5)	223 (16.2)	134 (9.7)	50 (3.6)
**Humiliation**	956 (69.3)	230 (16.7)	131 (9.5)	62 (4.5)
**Anxiety**	426 (30.9)	432 (31.3)	364 (26.4)	157 (11.4)
**Tension**	614 (44.5)	353 (25.6)	272 (19.8)	140(10.2)
**Exciting And Restless**	701(50.8)	371 (26.9)	214 (15.6)	93 (6.8)
**Confusion**	843 (61.1)	288 (20.9)	179 (13)	69 (5)
**Affect Liability **	472 (34.2)	339 (24.6)	294 (21.3)	274 (19.9)
**Eluding From People**	753 (54.6)	277 (20.1)	223 (16.2)	126 (9.1)
**Marked Anger**	655 (47.5)	346(25.1)	225 (16.3)	153 (11.1)
**Irritability**	561 (40.8)	361 (26.2)	287 (20.8)	170 (12.3)
**Increased Conflicts**	730 (52.9)	302 (21.9)	237 (17.2)	110 (8)
**Decreased Interest In Usual Activities**	443 (32.1)	363 (26.3)	299 (21.7)	274 (19.9)
**Concentration Difficulties**	712 (51.6)	323 (23.4)	243 (17.6)	101 (7.3)
**Lethargy**	270 (19.6)	462 (33.5)	392 (28.4)	255 (18.5)
**Fatigability**	290 (21)	126 (33.2)	397 (28.8)	234 (17)
**Impatience**	735 (53.3)	327 (23.7)	193 (14)	124 (9)
**No Control On Behavior**	765 (55.5)	308 (22.3)	197 (14.3)	109 (7.9)
**Total Psychological Symptoms**	43 (3.1)	803 (58.2)	452 (32.8)	81 (5.9)

When completing the forms, 55.9% of participants were in follicular phase and 44.1% experienced luteal phase of menstrual cycle. The reported frequencies of both physical and psychological symptoms among the students, who were in luteal phase, were a little higher than those in follicular phase but this difference was not statistically significant. In other words there was no association between PMS scores and the date of menstrual cycle at the time of filling out the forms (P = 0.846).

**Table 3:** . Percentage of the Sample Reporting Various Premenstrual Physical Symptoms from the Premenstrual Assessment Scale (n = 1379)

Item	No symptom, No (%)	Mild, No (%)	Moderate, No (%)	Severe, No (%)
**Lack Of Energy**	477 (34.6)	405 (29.4)	315 (22.9)	182 (13.2)
**Appetite Change**	636 (46.1)	340 (24.6)	250 (18.1)	154 (11.2)
**Overeating**	1067 (77.4)	145 (10.5)	123 (8.9)	44 (3.2)
**Food Cravings**	1019 (73.9)	132 (9.5)	116 (8.4)	112 (8.1)
**Hypersomnia**	617 (44.7)	344 (24.9)	266 (19.3)	152 (11)
**Insomnia**	946 (68.6)	225 (16.3)	128 (9.3)	80 (5.8)
**Breast Tenderness**	974 (70.6)	214 (15.5)	124 (9)	67 (4.8)
**Breast Swelling**	1111 (80.5)	124 (9)	94 (6.8)	50 (3.6)
**Flatulence**	812 (58.9)	263 (19.1)	178 (12.9)	126 (9.1)
**Swelling of Extremities**	1223 (88.7)	104 (7.6)	37 (2.7)	15 (1.1)
**Weight Gain**	1037 (75.2)	189 (13.7)	107 (7.8)	46 (3.4)
**Headache**	677 (49.1)	368 (26.7)	232 (16.8)	102 (7.4)
**Back Pain or Lower Abdominal Pain**	142 (10.3)	261 (18.9)	415 (30.1)	561 (40.7)
**Joint or Muscle Pain**	627 (45.5)	312 (22.7)	272 (19.8)	168 (12.1)
**Total Physical Symptoms**	23 (1.7)	1030 (74.7)	311 (22.6)	15 (1.1)

No correlation was also found between the presence of PMS/ PMDD and accommodation in dormitories (P = 0.637, P = 0.607), marital status (P = 0.601, P = 0.717) and amount of menstrual bleeding (P = 0.652, P = 0.242). On the other hand, there was statistically significant correlation between PMS/PMDD scores and variables including: age, mean score of educational performance, menarche age and weight. The correlations ranged from a high of 0.173 to a low of 0.056. Mean score of educational performance and menarche age were negatively related to PMS scores. We used multiple regression analysis to determine how well the combination of the four independent variables (age, mean score of educational performance, age of menarche and weight) explains the variance in the PMS scores. The hierarchical regression of PMS scores on four predictor variables entered in two blocks accounted for 4 % of variance and was significant at the 0.001 level. In the final model, age of menarche and educational performance mean score were significantly related to PMS scores. The negative relationship indicated that students who experienced menarche at earlier years, scored higher on PAS (Table 4).

**Table 4.** Multivariate Regression Analyses of the Dependent Variable PMS/PMDD Scores against Demographic and Menstrual Variables

	B	Beta	95% CI	
			Lower	Upper
**Educational performance mean score ^a^**	-1.505	-0.177	-1.995	-1.015
**Menarche age ^a^**	-1.639	-0.109	-2.502	-0.776

^a^ P < 0.001; R2 = 0.041; adjusted R2 = 0.39; B: un-standardized regression coefficients; Beta: standardized regression coefficients; CI: confidence interval.

Logistic regression for assessing the impact of dysmenorrhea on PMS showed that it made a significant contribution (P < 0.001). The odds of being afflicted with PMDD was 4 times higher for those who experienced dysmenorrhea (OR = 3.7, 95 % CI = 2.634- 5.078, P < 0.001). In the case of PMS, the odds of affliction was 13 times higher for those who reported dysmenorhea (OR = 13.129, 95 % CI= 2.469-69.7, P = 0.002).

## 5. Discussion

This study is the first report about the prevalence of premenstrual symptoms in Iranian adolescents based on the Premenstrual Assessment Scale (PAS), a culturally adaptive assessment tool. According to this scale, approximately 99.5% of the adolescent girls in the study population reported at least one mild to severe premenstrual symptom and met the criteria for PMS diagnosis and 59% had PMDD. This prevalence is higher than the one reported in other Iranian studies (54.7% to 98.2 %) but the comparison of results does not seem to be rationale.(18, 19, 22, 23) The scale used for assessment of premenstrual symptoms in these studies were just word by word translated forms of foreign assessment tools without making them compatible with the Iranian cultures. It is obvious that modifying and measuring the validity and reliability of any assessment tool is an integral part of any research.

The rates of PMDD and PMS in our study were also significantly higher than those in other countries ranging from 1.3% to 31%.4,(24-26) Issa and his colleagues also reported that 36.1% of the Nigerian medical students were suffering from PMDD.(27) The results of Steiner, Macdougall and Brown’s research was in line with reported prevalence rates from large prospective studies. They developed the Premenstrual Symptoms Screening tool" (PSST) which reflected a translated categorical DSM-IV criteria into a rating scale with degrees of severity.(28).

The precise causes of the difference in the prevalence of PMDD and PMS between the present study and other countries are still unknown, but we suggest several possible explanations. First, because psychiatric disorders assessment is based on respondent reporting, the reported prevalence is affected by cultural differences.(29) Second Iranians adolescents’ different life styles can be another explanation to these differences. Poor nutrition resulted from low income and low physical activity especially in adolescents accommodated in school dormitories may contribute to these differences. Third, women who report significant life stresses are more likely to rate premenstrual symptoms as severe and, therefore, be classified as having premenstrual disorder.4 Even though we did not address this factor but it seems that in the traditional society of Sabzevar, the potential role of high stress levels may also contribute to premenstrual disorder and exacerbate the symptoms such as depression, anger, tension and irritability. Considering the overall potential causes of PMS/PMDD, it seems logic that life style be the major factor influencing PMS/PMDD in Iranian population (30). Of additional interest was the number of some characteristics that assumed importance with respect to PMS and PMDD. These included educational performance, chronological age, age at menarche and dysmenorrhea. The present results indicated that those with dysmenorrhea were more likely to be classified as having PMS and PMDD. Furthermore, higher scores on PAS belonged to those who experienced menarche at earlier years and their educational performance mean scores were lower. This finding is consistent with the work of other investigators who reported that PMS is more prevalent in those who experience menstruation at early years of the adolescent period.(4) Whether the higher prevalence of premenstrual disorders in present study reflects a specific biological, social, or lifestyle characteristic remains to be determined by further studies.

This study also had a few limitations. One major limitation of our study was that we screened PMS/PMDD by retrospective self-report. According to the DSM-IV criteria, the diagnosis of PMDD requires the prospective daily charting which has to be completed over a period of at least two consecutive menstrual cycles. In a retrospective design of PMDD, women are likely to recall the worst episode in the past, so our questionnaire might select a broader group of women and had overestimated PMS and PMDD prevalence. Symptom daily rating can also be an impediment to study involvement. In this study only 1.5 % of the students completed the daily ratings of premenstrual symptoms based on PAS so we did not include them in analysis. Sternfeld and et al also reported that 30% of women refused to participate in data collection and only 50% of the enrolled women completed two cycles of daily ratings.(31) The main cause of this refusal reported by the present study sample was that the prospective daily charting was a time consuming process which interfered with the participants’ school homework so it seems logic that for this study sample, retrospective screening of PMS and PMDD should be preferably considered as a useful technique.

Another limitation is the generalizability of the results for all Iranian adolescents. Sabzevarian students may not be the representation of all Iranian adolescents. It is suggested that PAS be evaluated as a screening tool in different Iranian subpopulations. This study also indicated that the date of menstrual cycle has no effect on the self-report of premenstrual symptoms and no exacerbation will appear. However the major concern of this study is the high estimated rate of PMDD and PMS in a way that about 6900 Sabzevar high school students aged between 14 and 19 years currently suffer from premenstrual disorders and remain untreated.

In summary, this study indicated that premenstrual disorders are common in Sabzevarian adolescents and some factors including age, dysmenorrhea, age at menarche and educational performance are associated with premenstrual disorders. The new developed Premenstrual Assessment Scale (PAS) will contribute to further investigations of PMS and PMDD in Iranian adolescents. Preventive and treatment strategies for premenstrual disorders are also highly recommended.
